# Check the status of the development of children under age 5 in rural areas of Isfahan using the ASQ questionnaire in 2012-2013 year

**Published:** 2015

**Authors:** R Toghyani, F Sharafi Shorabi, H Sharafi Shorabi, SH Ghahraman Tabrizi

**Affiliations:** * Isfahan University of Medical Sciences, Isfahan, Iran

## Abstract

**Introduction:** Less than five years old child is gold time for a child for acquiring excellent skills such as perception, interaction, speech, etc., and therefore, the development of screening in this age to identify developmental problems in children early and provide treatment. To this order, the study intended to assess the development of children with a questionnaire ASQ in kids below five 5 year old in rural areas in Isfahan in 2012-2013.

**Materials and Methods:** A descriptive study was conducted on 1900 children under five years old, by separating year, one to two years, and 2 to 5 years in rural areas of Isfahan. The research sample included all children less than five years old were visited health coverage for care. Exclusion criteria for selecting children less than five years was birth defects, such as heart disease, kidney disease. The sampling method was an interview with the mother standardized questionnaire the World Health Organization and the Ministry were used to collect data by in the field of evolutionary 5 (Gross and fine motor, issue solving and interaction, personalization and social). There are six questions in per area, and if yes score 10 and sometimes score 5 and if it still does not do the activity score is zero. The questionnaire delivered to the mother and then returned to where it was necessary to complete the application in question and data was analyzed SPSS21.

**Findings:** Findings indicated that the average age of moms via a mean age of 5.5 ± 28.8 of children 1.4 ± 22 months. 95% of mothers were homemakers, and 33.1 percent had primary education. Regarding the evolution of the most challenging year in the development of gross motor 4.5 and then the solution was 3.7. In group most difficult one to two years of growth in gross motor and fine to the amount of 4.5 to 3.7 after the problem was and in 2 to 5 years in most areas of fine motor problem solving with 7.2 and then 2.8, respectively.

**Discussion:** According to the findings shown first on gross motor developmental problems at an early age is the greatest and whatever increasing age of the more advanced developmental problems such as fine motor goes. On the other hand, problem solving in children less than one year of age accounted for the highest frequency, which indicates that both service providers and parents In the field of education are not necessary. It should consider the policy of this country and as mothers walking, talking, etc. to teach children skills such as problem solving in the field of education to teach children.

## Introduction

The death index of children under five years due to various social and economic factors that increase or decrease reflects the development of countries. To this end, in 2000, countries pledged to reduce the index by 50% by 2015 [**1**]. One of the strategies is reviewed the history of logic in child care that countries with a systematic program. This process has three stages, and at the end of each stage should be thinking about planning for the next stage. Firstly, according to child survival, the more children trying to survive. Secondly, due to the physical growth of the child, the surviving children must be the indices of growth desired standard and the third stage regarding the development of children, the diagnosis, and early intervention developmental disorders [**2**,**3**].

Growth is the increase in the number of cells that have already formed or enlargement and the gradual increase in the size of the body, organs, and tissues from conception to childhood, adolescence to adulthood and evolution means the ability, and skills and change from pre-birth to adulthood [**3**]. Under five years of age is critical in the development of children in this age period, the following year is of particular importance. This stage of life is a significant step in the process of growth and development, they are irreversible, lack of attention to each of the risk factors causes severe complications, impaired growth, and development in children and increases the likelihood of complications and mortality [**4**]. Therefore, assessment of child development should be part of routine examinations of healthy children, or in other words the development of screening. Research shows that 70 percent of doctors do a screening tool for the development of children [**3**].

On the other hand, one of the problems of developing countries, including Iran, in spite of progress in improving maternal and infant health promotion high levels of high-risk pregnancies (Pregnancy at an early age, pregnancy and small distance, lack of access to health centers, lack of facilities safe delivery, gestational age over 35 years old, unwanted pregnancy, the presence of certain diseases in mothers, lack of consultation before pregnancy). Performing unnecessary C-section before the deadline and increase the population of infants at risk of developmental disorders in medical and environmental [**3**,**4**]. Also, it should also be noted that the improvement of health care in the neonatal intensive care unit and access to treatment and the proper equipment to increase the survival of these babies and consequently the likelihood of irreparable physical and mental effects of infancy, childhood and school-age and older increased [**5**,**6**].

As mentioned earlier golden age for the development of children is under five years to say his skills as understanding, mutual communication, gross motor, fine motor, speech, etc. and development of children in this age screening to identify developmental problems and provide treatment [**6**,**7**]. Investigations in 2004 showed that only 30 percent of developmental problems in children without any interference detected at the right time, this is while if the use of a screening tool compared to a 70% [**7**]. So firstly, the use of a screening tool that has a high sensitivity and specificity and on the other hand, the criteria of low cost, easy to use and accessible, it is necessary, and secondly, the golden child under five years should be to check the status of development [**3**,**7**].

Since the study of the evolution of the situation of children in recent years as one of the priorities identified by the World Health Organization in Iran, concerning programs that reduce mortality and improve the health and growth indicators, more attention to the promotion of child development is inevitable. Therefore, since 2012 as a priority in child health and developmental screening for children and aims to be the definitive diagnosis of children with developmental problems. To identify problems and determine effective interventions to improve the program while the program is imperative that an effective strategy, applied research in this direction. Studies show that many studies have been done, especially in Iran. Therefore, this study aimed to determine the evolution of the situation of children in the age group of under one year, one to two years, and two to five years. So that using standard tools (questionnaires ASQ*) of child development and implementation of effective interventions identified and designed to improve the health status of children is a step to be taken.

**Materials and Methods**

A descriptive study on 1900 children under five years of age, by the following year, one to two years and 2 to 5 years in rural areas of Isfahan. Health houses covered research sample of all children under age five. To determine samples from a simple random sampling method, were used that classified each category was considered a health center. Given that there are about 540 health centers in Isfahan province, home health cover at least 20 children under age five were selected. Then in each health center through the Office of children Care, children under five years who regularly visit and are covered by any particular disease (such as metabolic disease), refractory and had no abnormalities, Were randomly selected and invited to the mother, explanations should refer to him. Then calculate the exact age of the child at the same age and a questionnaire completed by the mother in the presence of the questioner on the same day. If the mother had problems responding to some questions, after learning of his mother’s questionnaire within a week survey mothers completed and handed over to the questioner.

Ages and Stages Questionnaires can complete by parents and careers of the child standard questionnaire with high sensitivity and specificity (Respectively 83 and 90 percent), given that development of the child or the skills that your child’s life. The child’s age is a function of the questionnaire, accordingly designed and skills in five areas (communication areas, gross and fine motor, individual, social and issue solving) were checked. 19 surveys ranging age of 4 up 60 months and up, until the age of 2 years, two months, 2 to 3 years of age every three months and 3 to 6 years old, completed questionnaires every six months. In section 6 questions to each of the questions, there are three options. If the child is doing the activity, yes and score 10 and if he is capable of doing at times, and sometimes the option 5 and if the lack of action at the moment, options not yet given a zero score.

Total score in each area specified by standard pre-determined cut-off points of comparison will be based on compliance and norms. If the total rated in the top of the column was one standard deviation, the child had no developmental problems, between one and two standard deviations. The need for further investigation and, if the child is below two standard deviations of the rating, child developmental problem that all children in these situations referred to specialized centers for further diagnostic workup. Questionnaires ages and stages of development on a global scale is a valid instrument to the validity of the 0.88 – 0.83 and reliability 0.90 – 0.94, compliance and normalization in Iran in 2006 in accordance with the Ministry of Health University of Social Welfare and Rehabilitation. UNICEF conducted and validated 0.84 and reliability 0.94 and 96 percent reported the ability of the test to determine the developmental disorder.

Completed questionnaires were recorded and analyzed in the software SPSS 21. Exclusion criteria for children selected less than five years of birth defects, such as heart disease, kidney disease.

**Findings**

Results showed that the mean age of 5.5 ± 28.8, and most women 30 to 34 years was 24.6 percent. The average age of children was 1.4 ± 22 months. For most children in the 2 to 4 percent of children with the 55.8 and 41.3% of the mothers had one child, 33.1% of mothers of primary education, 95.2 percent were homemakers. Most of the employed father of the 40.4 percent. 37 percent to 40 weeks gestational age 75.9 and 59.8 percent were caesarean section. Terms the situation evolution, the most challenging year in the group under development of gross motor 4.5 and then the solution was 3.7 and the lowest frequency to communicate with 0.9 percent (**Table 1**). In the age group most difficult one to two years of development of communication and fine motor with the 4.5 per cent and after it problem solving with the amount of 3.7 per cent and the minimum frequency to the realm of personal, social, or 2.3 percent (**Table 2**). In the age group of two to five years, most evolutionary problems related to the scope of the poblem with the amount of 7.2 percent and after that at a rate of 8.2 percent, the lowest frequency of fine motor gross motor with 10.8 percent (**Table 3**).

**Table 1:** Distribution of absolute and relative status of children less than one-year-old home health development of the province according to the development in 2012

Standard deviance above one		Between 1 and 2		Standard deviance below one		Area
Percent	Number	Percent	Number	Percent	Number	
92.8	597	6.2	40	0.9	6	Communicate
89.3	574	6.2	40	4.5	29	Coarse movement
91.0	585	5.7	37	3.3	21	Fine Motor
90.8	584	5.5	35	3.7	24	Problem-solving
87.2	561	10.0	64	2.8	18	Personal and social

**Table 2:** Distribution of absolute and relative frequency of development of children two to five years referred to Isfahan health house separate areas for development in 2012

Standard deviance above one		Between 1 and 2		Standard deviance below one		Area
Percent	Number	Percent	Number	Percent	Number	
83.7	481	11.8	68	4.5	26	Communicate
91.5	526	4.8	28	3.7	21	Coarse movement
83.7	481	11.8	68	4.5	26	Fine Motor
87.7	504	8.0	46	4.3	25	Problem-solving
93.5	538	4.2	24	2.3	13	Personal and social

**Table 3:** Comparison between the means of seminary and Allameh’ students regarding other variables

Standard deviance above one		Between 1 and 2		Standard deviance below one		Area
Percent	Number	Percent	Number	Percent	Number	
92.3	629	0.4	37	2.3	16	Communicate
90.6	618	7.6	52	1.8	12	Coarse movement
89.0	607	8.2	56	2.8	19	Fine Motor
79.9	545	12.9	88	7.2	49	Problem-solving
92.7	632	5.1	35	2.2	15	Personal and social

## Discussion

The results of developmental problems in 5 regions (interaction, gross and fine motor, issue-solving, personal and community) and in the age groups of under one year, one to two years, and two to five years reviewed. Firstly, results showed that the lowest age under one year of age divided into age groups, most problems related to gross motor development is the increasing age of the more advanced developmental problems such as fine motor and problem solving. On the other hand, the results show that problem solving in children less than one year of age most frequent developmental problems accounted for perhaps one of the reasons, is that the skills to the children of parents in education are small and, therefore, their education will be less. Many factors, including genetic factors, environment, and education level of parents, social factors and influences on the evolution of children. Studies show that caregivers, parents, environment, community, and country programs, and policies have a significant impact on skills such as walking, speaking, understanding, etc. [**7**-**9**]. In fact, the experiences of children in the first five years of their lives is a framework to enter the later stages of his life [**7**,**9**].

As already mentioned, one of the factors that raise awareness and change attitudes and practices of parents in childcare and development and affect their growth and development, health care workers and doctors. For example, most parents now about the interaction (the area) are training and educating their children at home. In the present study the children in this area almost desirable [**10**]. Also, a child younger than two years of development work is faster and why families are more sensitive to their child at home and labor environment and in the case of delay in development, the sooner they realize their action [**11**]. In a study was conducted by Dorothy in 2002 results showed that many children skills such as movement, language, speech, understanding, and the acquisition is the first in 4 years. That is to say; the period is as a golden age for early child development (early child development) [**12**].

In research by Shahshahani et al. (2008) in 197 children aged 4 to 60 months in Tehran on using some of Denver and ASQ questionnaire used, the results showed that most developmental problems in fine motor and gross motor rate of 5%, after the 3.6 percent rate calculated [**13**]. In a study by Ali Akbari and colleagues the results showed in 2009 in Isfahan most developmental problems in children 4 to 60 months of age at a rate of 7.2 percent for fine motor and personal and social areas with the lowest prevalence of 1.7 percent [**14**]. In a study by Cross to check the status of children born to teenage mothers in England was developed. The results showed that of 45 patients who had completed the ASQ form, the most frequent problem is related to the amount of 11.1% (5 cases), and the lowest rate was in the area of interaction. (2.2%) (1 case) [**15**].

In a cohort study on 4546 children under the age of 36 weeks in 2002 and 2003. The questionnaire completed ASQ; the results showed that the greatest problem with the development of fine motor 4.89 percent and the lowest frequency of interaction with the 4.05 percent. Results of studies of the present study are consistent with almost [**16**]. In a research via Soleimani on 250 mothers of kids under 12 months old in the city of Qazvin was conducted in 2011 showed the maximum prevalence of developmental delay in the areas of association (8%) and the minimum prevalence of issue with the amount of 2 percent [**17**]. In a study on 114 children aged 4 to 60 by Darreh et al. month of hospitalization in intensive care and the average age of 17 months have been the most difficult development according to ASQ questionnaire related to communication (20.2%) and the lowest frequency has been related to the problem (8.8%) [**18**].

The difference between these studies, the present study is that the first developmental problems have not determined according to age groups, but in this study, as this age group Shahshahani gross motor and fine motor under one year of age had the highest rate of one in two years. Secondly, scale detection of developmental problems in these studies is not known. In the study Darreh, according to the percentages obtained is two standard deviations considered as a developmental problem or below two standard deviations between his two standard deviations found. In general, in the studies, the prevalence of disability in children with high risk varies due to changing criteria of the study, combined study population, the duration and can be followed and assessment tools.

According to the cases mentioned, planning for family education and skills training for children, justification educators, and caregivers in various areas of development and teaching of children, studies on the impact of the acquisition of expertise training to families.

## References

[1] lornejad H (2009). Military reported the deaths of children in care, 59-1 months. Ministry Hygiene Remedy and medical education health family office child health Operation.

[2] Karimzadeh P (2008). Development and child development disorder.

[3] Barakati H (2013). Child development screening. Ministry Hygiene Remedy and medical education health family office child health Operation.

[4] Guidelines optimal feeding of low birth weight infants in low –and middle-income countries on. Available at: URL: http//www. Who.int.

[5] Sajedi F Development and childhood developmental problems. Available at: URL: http//www. /www1.jamejamonline.ir.

[6] Soleimani F (2007). Developmental outcome of LBW and premature Infants. Iran J Pediatr.

[7] Talaei G (2011). Growth and development guideline. Ministry Hygiene Remedy and medical education-family health office-child health.

[8] Early child development: a powerful equalizer. Available at: URL: http//www. Who.int.

[9] Karimi M, Fallah R, Dehghanpoor A, Mirzaei M (2011). Developmental status of 5-year-old moderate low birth weight children. Brain Dev.

[10] (2005). Well Child Car. Ministry Hygiene Remedy and medical education health family office child health Operation.

[11] Soleimani  F (2001). Motor Developmental Delay Screening in Iranian Infants. Brain & Development.

[12] Doherty  G Graph developed by Council for Early Child Development. 1997 Human Resources Development Canada 2000..

[13] Shahshahani Pour  S, Vameghi  R, Sajedi  F, Hatamizadeh  N (2006). Early detection, diagnosis and an introduction to early intervention in childhood developmental problems.

[14] Amir Ali Akbari  T, Orabi  FS, Amiri  S, Soleimani  F, Alavi Majd  H Correlation between high-risk pregnancy and developmental delay in children aged 4–60 months. Libyan J Med.

[15] Ryan-Krause  P, Meadows-Oliver  M, Sadler  L, Swartz  M Developmental Status of Children of Teen Mothers: Contrasting Objective Assessments with Maternal Reports.

[16] Kerstjens  JM, Bos  AF, Ten Vergert  EM, de Meer  G, Butcher  PR, Reijneveld  SA (2009). Support for the global feasibility of the Ages and Stages Questionnaire as a developmental screener. Early Hum Dev.

[17] Solimani  F, Bajelan  Z, Amir Ali Akbari  S, Alavi Majd  H (2013). Correlation between Anemia during Delivery and Developmental Delay in Children 12 Months in Qazvin 2011-2012. Pediatric Neurorehabilitation.

[18] Dorre  F, Bayat  F (2011). Evaluation of children’s development (4-60mo) with a history of NICU2011. Journal of Ardabil University of Medical Sciences & Health Services.

